# CACTUS: integrating clonal architecture with genomic clustering and transcriptome profiling of single tumor cells

**DOI:** 10.1186/s13073-021-00842-w

**Published:** 2021-03-24

**Authors:** Shadi Darvish Shafighi, Szymon M. Kiełbasa, Julieta Sepúlveda-Yáñez, Ramin Monajemi, Davy Cats, Hailiang Mei, Roberta Menafra, Susan Kloet, Hendrik Veelken, Cornelis A.M. van Bergen, Ewa Szczurek

**Affiliations:** 1 Faculty of Mathematics, Informatics, and Mechanics, University of Warsaw, Stefana Banacha 2, Warsaw, 02-097 Poland; 2 Department of Biomedical Data Sciences, Leiden University Medical Center, Einthovenweg 20, Leiden, 2333 ZC The Netherlands; 3 Department of Hematology, Leiden University Medical Center, Albinusdreef 2, Leiden, 2333 ZA The Netherlands; 4 Leiden Genome Technology Center, Leiden University Medical Center, Einthovenweg 20, Leiden, 2333 ZC The Netherlands

**Keywords:** Single-cell sequencing, Follicular lymphoma, B cell receptor, Clonal evolution, Somatic mutations, Probabilistic graphical model

## Abstract

**Background:**

Drawing genotype-to-phenotype maps in tumors is of paramount importance for understanding tumor heterogeneity. Assignment of single cells to their tumor clones of origin can be approached by matching the genotypes of the clones to the mutations found in RNA sequencing of the cells. The confidence of the cell-to-clone mapping can be increased by accounting for additional measurements. Follicular lymphoma, a malignancy of mature B cells that continuously acquire mutations in parallel in the exome and in B cell receptor loci, presents a unique opportunity to join exome-derived mutations with B cell receptor sequences as independent sources of evidence for clonal evolution.

**Methods:**

Here, we propose CACTUS, a probabilistic model that leverages the information from an independent genomic clustering of cells and exploits the scarce single cell RNA sequencing data to map single cells to given imperfect genotypes of tumor clones.

**Results:**

We apply CACTUS to two follicular lymphoma patient samples, integrating three measurements: whole exome, single-cell RNA, and B cell receptor sequencing. CACTUS outperforms a predecessor model by confidently assigning cells and B cell receptor-based clusters to the tumor clones.

**Conclusions:**

The integration of independent measurements increases model certainty and is the key to improving model performance in the challenging task of charting the genotype-to-phenotype maps in tumors. CACTUS opens the avenue to study the functional implications of tumor heterogeneity, and origins of resistance to targeted therapies. CACTUS is written in R and source code, along with all supporting files, are available on GitHub (https://github.com/LUMC/CACTUS).

**Supplementary Information:**

The online version contains supplementary material available at (10.1186/s13073-021-00842-w).

## Background

Tumor heterogeneity and clonal evolution present a major challenge for cancer therapy [1]. Tumor cells carry founder and subsequently acquired driver mutations that cause transformation of the healthy cell into an expanding population of malignant cells. Continuous acquisition of mutations creates populations of tumor cells with divergent mutational profiles. Diverging cells with acquired driver mutations result in preferential clonal expansion leading to intraclonal diversity. Given that distinct genotypes induce key phenotypic differences between the clones [2], gene expression variation between the clones is expected. Measuring the phenotypes of tumor clones, however, is challenged by the difficulties in resolving the clonal genotype-to-phenotype maps in tumors [3].

Follicular lymphoma (FL) is a common type of malignant B cell lymphoma with characteristics of normal germinal center (GC) B cells. FL cells maintain the typical follicle-like structure of normal GC reactions in response to pathogens. FL pathogenesis is founded by the paradigmatic translocation (14;18)(q32;q21) that places BCL-2 under transcriptional control of the IGH@ locus enhancer. Secondary drivers affect genetic modifiers that enhance germinal center (GC) formation, reduce B cell differentiation, and freeze FL cells in the GC stage [4, 5]. Despite commonly observed pathogenic genomic events, clinical behavior of FL is unpredictable and ranges from spontaneous remission over long-term stable disease to transformation to aggressive B cell lymphoma.

In addition, FL cells are continuously exposed to a physiological mutator mechanism, i.e., expression and action of activation induced cytidine deamidase (AID) [6]. AID focuses on B cell receptor (BCR) loci and results in highly mutated BCR heavy and light chain genes in FL [7]. Whereas BCR mutations intrinsically may lead to a proliferative signal by acquisition of N-linked glycosylation [8], preferential expansion of clones with identical BCR can also be explained by co-acquisition of underlying driver mutations that enhance their proliferation. In addition to grouping of individual cells into evolutionary clones by exome-wide mutations and structural variants, single FL cells can also be clustered based on the expression of identical BCR sequences. BCR mutations can therefore be considered events in clonal evolution in FL and present suitable markers that may allow a more accurate reconstruction of clonal evolution than based on exome mutations only.

Elucidation of tumor evolution and reconstruction of the tumor clonal architecture are possible from bulk DNA sequencing [9–12] and from single-cell (sc) DNA sequencing data [13–16]. The outcome of such evolutionary analysis is a set of tumor clones, defined by their genotypes and frequencies. The genotype indicates which mutations are present in each clone, and the frequency indicates the fraction of cells from that clone in the entire tumor cell population. The task of identifying the tumor clones and their genotypes is computationally very difficult [12], and thus, the tumor clone genotypes inferred from DNA sequencing alone are likely to be imperfect.

Recent efforts into the direction of mapping genotypes to phenotypes in tumors include characterizing gene expression profiles of tumor clones based on matching the single-cell RNA sequencing (scRNA-seq) readouts to copy number variants in the clones [17–19]. Poirion et al. [20] proposed a linear model detecting association of single nucleotide variants from scRNA-seq with gene expression. This approach, however, ignores the evolutionary history of the tumor, which can be resolved to determine the genotypes of the tumor clones. Such obtained genotypes can then be matched to mutations observable in scRNA-seq. Recently introduced cardelino [21] is the first approach to successfully utilize the mutation mapping between the clone genotypes and the variants in scRNA-seq data. The performance of this approach, however, can be hampered by the fact that single-cell transcripts contain only information on 5 ^′^ part of the RNA and that the data are sparse. With such limited data, the confidence of assigning single cells to clones, and thus also of clonal genotype to gene expression phenotype mapping, is also limited. Here, we define the confidence as the concentration of the probability distribution of the cell-to-clone assignment, with high confidence corresponding to a high probability of assignment to one clone and low confidence corresponding to a uniform probability over clones. To increase the confidence, additional available evidence should be integrated into the inference. One such evidence is a given clustering of cells, such as the grouping of cells by their similar BCR sequences in FL evolution. Combining multiple data sources has the potential to increase the resolution of tumor heterogeneity analysis [22], but is computationally challenging [23] and calls for a dedicated probabilistic model.

Here, we propose a probabilistic graphical model for integrating Clonal Architecture with genomic Clustering and Transcriptome profiling of single tUmor cellS (CACTUS). The model extends cardelino [21] and maps single cells to their clones based on comparing the allele-specific transcript counts on mutated positions to given clonal genotypes, leveraging additional information about evolutionary cell clusters. As part of the model inference, CACTUS corrects the input clone genotypes and adjusts the input cell clustering using all available data. The input clusters should be defined based on additional evolutionary information, in such a way that the model can assume that cells in the same cluster tend also to belong to the same tumor clone.

**Fig. 1:**
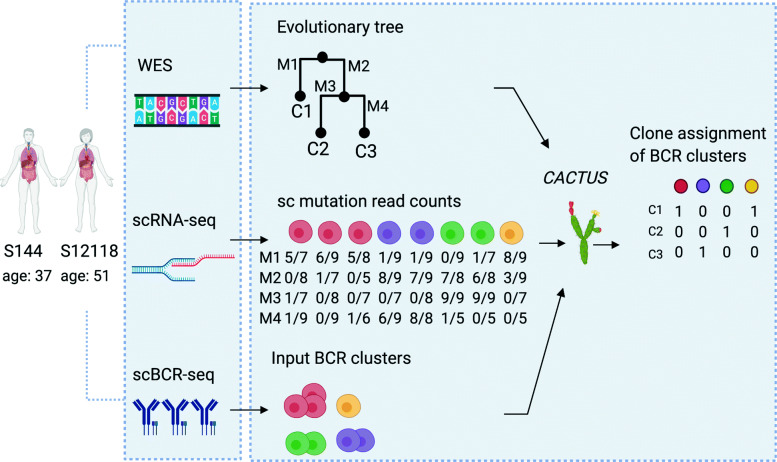
Overview of the patient data analysis and the CACTUS model. Whole-exome sequencing and single-cell sequencing of all transcripts, as well as single-cell sequencing of BCR, were performed on samples from two FL patients. Using WES, imperfect clonal evolution could be inferred and given as a prior to the model (C1, C2, …). From scRNA-seq, allele-specific transcript counts (mutated/total) were extracted at mutated positions (M1,M2, …). Input BCR clusters were defined as clusters of cells with identical BCR heavy chain sequences. The data of input tumor clones, mutation transcript counts, and given single-cell clusters (here, the BCR clusters) are combined in the CACTUS model for inference of the clonal assignment of the clusters. Both the input clone genotypes and clustering are considered potentially imperfect and are corrected during the inference using all available data. Image created with Biorender.com

We apply CACTUS to newly generated whole-exome sequencing (WES), scRNA-seq, and single-cell BCR sequencing data of FL tumor samples from excised malignant lymph nodes of two subjects. As a result, the single cells are assigned to their clones of origin, accounting for the similarities of their BCR sequences (Fig. 1). We demonstrate that guided by the BCR sequence information, CACTUS assigns single cells to tumor clones in agreement with independent gene expression clustering. For both subjects, CACTUS maps cells and BCR clusters with substantially higher confidence than cardelino. These results indicate that the important challenge of tumor genotype-to-phenotype mapping can successfully be approached by probabilistic integration of multiple measurements.

## Methods

### Follicular Lymphoma sample preparation

Samples with histologically confirmed infiltration of follicular lymphoma were collected with approval by the institutional review board of Leiden University Medical Center according to the Declaration of Helsinki and with written informed consent. Single-cell suspensions were obtained by gentle mechanical disruption and mesh filtration and were cryopreserved using 10% DMSO as cryoprotectant. The remaining tissue was cultured in low-glucose DMEM to obtain stromal cell cultures for isolation of DNA of non-malignant cells. Thawed single FL cells were purified by flow cytometry using fluorescently labeled antibodies specific for CD19 and CD10 and rested overnight followed by removal of dead cells using the MACS dead cell removal kit. Cells of different patients were pooled and loaded on a 10X Genomics chip to obtain single-cell cDNA libraries for an expected 1500 cells per patient. Following single-cell cDNA library generation and amplification, one fraction was directly sequenced for 5 ^′^ gene expression profiling. The second fraction was enriched for BCR transcripts by seminested amplification using 3 ^′^ constant domain primers for all BCR genes, partially digested and sequenced. Both single cell libraries were sequenced in paired-end mode on Illumina (2 × 150 bp).

### WES sequencing and mutation calling

FL single cells were purified by flow cytometry as described above to obtain bulk purified FL cells for immediate isolation of DNA. Whole-exome sequencing (WES) was performed on paired FL and normal DNA at 200 × and 50 × coverage, respectively. Genomic DNA was isolated using the QIAamp DNA Mini kit (Qiagen). Samples were sequenced (HiSeq 4000 instrument, Illumina Inc.) in paired-end mode on Illumina (2×101 bp) using TrueSeq DNA exome kit (v.6) (Illumina Inc.). Paired-end reads were aligned to the human reference genome sequence GRCh38 using BWA–MEM (V0.715-r1140) [24]. Deduplication and alignment metrics were performed using Picard tools (v2.12.1). Local realignment was performed around indels to improve SNP calling in these conflicting areas with the IndelRealigner tool. Recalibration to avoid biases was performed following the Genome Analysis Toolkit (GATK) Best Practices [25]. Single mpileup files were generated from paired bam normal/tumor using samtools mpileup (v1.6). Mutation calling and computation of somatic *p* values (SPV) was performed on mpileup output files using Varscan (v2.3.9)[26] to WES data from tumor and patient-matched normal samples with a minimum coverage of 10 ×. Quality control metrics were assessed using FastQC (v0.11.2)[27] before and after the alignment workflow and reviewed to identify potential low-quality data files.

### Single-cell data processing

Sequencing data was processed with 10X Genomics Cell Ranger v2.1.1 with respect to GRCh38-1.2.0 genome reference to obtain UMI-corrected transcript raw gene expression count tables, BAM files, and BCR all_contig.fasta files.

To generate single-cell allelic transcript counts, we used a custom-made script to identify reads intersecting with WES-based mutated positions. For each read, to classify the allele, we identified the single nucleotide overlapping the mutated base. To obtain transcript counts, we used the unique molecular identifiers (UMIs) associated with the reads.

We used the vireo function from cardelino package v0.4.2 to construct clusters of cells sharing the same germline genotype. As input, we provided allelic counts for the positions likely to differ between the subjects and not mutated between FL and stromal cells. For further processing, we selected cells assigned to a single subject at minimum probability threshold of 0.75. Once the clusters of cells sharing the same germline genotype were identified, we assigned them to patients by comparing the cluster consensus genotype with the patient-labeled genotypes obtained from WES.

IMGT/HighV-Quest [28] was used for high-throughput BCR analysis and annotation of the BCR all_contig.fasta file [28]. IMGT/HighV-Quest output data was filtered for productive and rearranged sequences, and FL cells with identical BCR heavy chains were considered unique BCR clusters within the malignant cell population and were annotated with unique identifiers. R-package “vegan” was used to calculate Pielou’s index of evenness for BCR cluster size distribution.

### Phylogenetic analysis

For each subject, we first identified common mutations that can be found in both WES data and scRNA-seq data. Next, we used FALCON-X with default parameters for estimation of allele-specific copy numbers from WES data. As a verification, we compared the results of FALCON-X with those of GATK CNV analysis pipeline, and confirmed that the two approaches gave similar results. Finally, we run Canopy [9], providing the estimated major and minor copy number, as well as the allele-specific read counts in the tumor and matched normal WES data as input. Taking advantage of a Bayesian framework, Canopy estimates the clonal structure of the tumor for a pre-specified number of clones. Choosing between trees with the number of clones from 2 to 4, for both subjects, the BIC criterion used by Canopy suggested trees with 4 clones as the best solution. For further analysis, for each subject, we selected the top tree returned by Canopy (see Additional file 1 for the posterior likelihood and BIC plots of Canopy for subjects S144 and S12118, respectively).

### Mapping BCR clusters to tumor clones using CACTUS

**Fig. 2:**
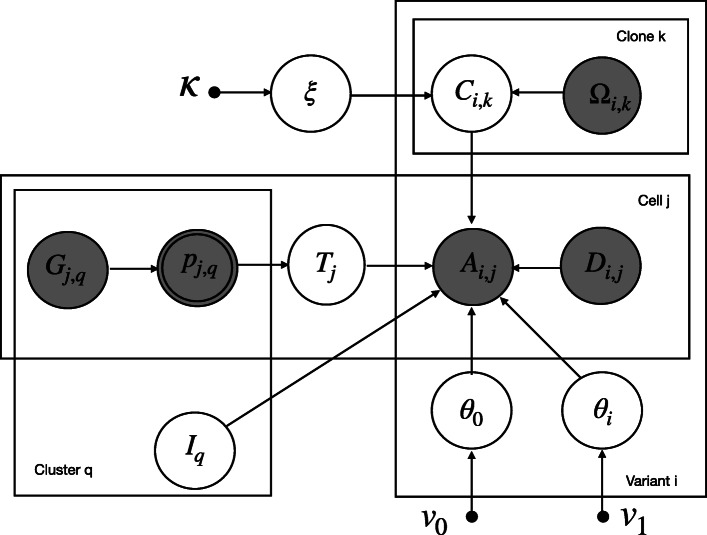
The graphical model representation of CACTUS. Circle nodes are labeled with random variables in the model. Arrows correspond to local conditional probability distributions of the child variables given the parent variables. Observed variables are shown as grayed nodes. Double-circled nodes are deterministically obtained from their parent variables. Small filled circles correspond to hyperparameters. *C*_*i*,*k*_ denotes the true (corrected) genotype of clone *k* at variant position *i*. *Ω*_*i*,*k*_ denotes the input clone genotypes, with *Ω*_*i*,*k*_=1 if the mutation *i* is present in clone *k* and 0 otherwise. *G*_*j*,*q*_ denotes the distance of the cell *j* to cluster *q*, computed based on the input clustering of cells. *T*_*j*_=*q* indicates that cell *j* is in cluster *q*. *p*_*j*,*q*_ is interpreted as the success probability for cell *j* to switch to cluster *q*. *A*_*i*,*j*_ denotes the observed count of unique transcripts with alternative (mutated) nucleotide mapped to position *i* in cell *j*. *D*_*i*,*j*_ denotes the total unique transcripts count mapped to that position in that cell. *I*_*q*_=*k* represents the assignment of cluster *q* to clone *k*. *θ*_*i*_ denotes the success probability of observing a transcript with the alternative nucleotide at a position *i* in a cell that carries this mutation, and *θ*_0_ the success probability of observing a transcript with the alternative nucleotide in a position that is not present in the cell. *ξ* is the error rate for the genotypes. {*ν*_0_,*ν*_1_,*κ*} constitutes the set of hyperparameters in the model

Below, we introduce a probabilistic model, CACTUS, for mapping a given set of cell clusters to tumor clones based on the mutation matching between the cells in clusters and the clone genotypes (Fig. 2). In this analysis, the input clusters corresponded to sets of cells with identical BCR sequences. The input clustering and input clone genotypes were corrected during the inference process, taking into account all available data. Both CACTUS and cardelino are inferred using Gibbs sampling. For each subject, CACTUS was run for the top Canopy tree for a maximum of 20,000 iterations of the Gibbs sampler, with 10 different starting points. For the sake of comparison, cardelino was applied with the same setup.

CACTUS is a direct extension of cardelino [21], accounting for cell clustering, with the assumption that cells in the same cluster belong to the same clone. Let *i*∈{1,…,*N*} index mutation positions, which can be identified both in bulk DNA sequencing and single-cell RNA-seq data (see above). We assume we are given at input a set of *K* tumor clones, indexed by *k*∈{1,…,*K*}. Each tumor clone is represented by its genotype and prevalence in the tumor population. The input clone genotypes are represented by a binary matrix *Ω*_*i*,*k*_ with entries equal 1 if the mutation *i* is present in clone *k* and 0 otherwise.

We are also given an independent clustering of single cells, where each cluster *q*∈{1,…*Q*} contains a number of cells and the clusters are assumed not to overlap. Let *j*∈{1,…,*M*} index cells. We assume that the input clustering is imperfect, and thus, we define the true (corrected) clustering by a set of hidden categorical variables **T**={*T*_1_,…,*T*_*M*_}, with each *T*_*j*_ taking values in {1,…,*Q*} and *T*_*j*_=*q* indicating that cell *j* is in cluster *q*. We assume a categorical distribution for *T*_*j*_:

$$P(T_{j} = q|p_{j,1},\ldots,p_{j,Q}) = p_{j,q}, $$

where $\sum _{q} p_{j,q}=1$. The parameters of the categorical distribution *p*_*j*,*q*_ are interpreted as the success probabilities for cell *j* to switch to cluster *q*. We assume these success probabilities are dependent on the input clustering of cells. Denote **p** the matrix with elements *p*_*j*,*q*_,**p**=(*p*_*j*,*q*_)_*j*=1,…,*M*,*q*=1,…,*Q*_. Let *G*_*j*,*q*_ denote the distance of the cell *j* to cluster *q*, obtained from the input clustering. Based on *G*_*j*,*q*_, the probability *p*_*j*,*q*_ is defined as:

$$p_{j,q} = \frac{e^{- cG_{j,q}}}{\sum_{q^{\prime}}{e^{- cG_{j,{q^{\prime}}}}}}, $$

where *c* is a constant determining the strength of the prior. This parameter should be defined by the user. Here, we set *c*=2. In this application, the input clustering is defined as sets of cells with identical BCR sequences. Therefore, each input cluster is represented by the shared BCR sequence of its cells. Based on such input clustering, for each cell *j* and cluster *q*, the distance *G*_*j*,*q*_ is computed as the number of different mutations between BCR sequence of cell *j* and the representative BCR sequence of cluster *q*. Thus, the distance of *q* to its own cluster equals 0. For cells which did not have its BCR sequenced, we set their distance to their own cluster to 0, and their distance to all other clusters as equal to the mean of all known distances of cells to clusters.

We are interested in assignment of the cell clusters to the clones. The clone assignment of each cluster *q* is represented in the model by a hidden variable *I*_*q*_ with values in {1,…,*K*}. We assume a uniform prior for *I*_*q*_ and set $P(I_{q}=k) = \frac {1}{K}$. Alternatively, the prior could depend on the prevalences derived from the evolutionary model. The probability of cluster-to-clone assignment returned by CACTUS is computed from the Gibbs sampling iterations, as the posterior probability distribution of *I*_*q*_. The single cells are assigned to each clone with the same probability as their cluster. Thus, for each cluster *q* and each cell *j* in *q*, the assignment probability of *j* to clone *k* equals the probability of assignment of *q* to *k*.

We assume that the input clone genotypes can contain errors with error rate *ξ*. The prior distribution for the error rate is parametrized by *κ*=(*κ*_0_,*κ*_1_) and is set to *P*(*ξ*|*κ*)=Beta(*ξ*;*κ*_0_,*κ*_1_). We define a hidden random variable *C*_*i*,*k*_ denoting the true (corrected) genotype of clone *k* at variant position *i*, with:

$$P(C_{i,k}=1|\Omega_{i,k},\xi) = \xi^{1-\Omega_{i,k}} \times (1-\xi)^{\Omega_{i,k}}.  $$

Let matrix **A** with elements *A*_*i*,*j*_ denote the observed count of unique transcripts with the alternative (mutated) nucleotide mapped to position *i* in cell *j*, and matrix **D** with elements *D*_*i*,*j*_ denote the total unique transcripts count mapped to that position in that cell. Let *θ*_*i*_ denote the success probability of observing a transcript with the alternative nucleotide at a position *i* in a cell that carries this mutation, and *θ*_0_ the success probability of observing a transcript with the alternative nucleotide in a cell that does not carry this mutation genotype of the cell. The distribution of the observed read counts then becomes:

$$\begin{aligned} P(A_{i,j}|D_{i,j},I_{q},C_{i,I_{q}},\theta, T_{j}=q) = \left\{ \begin{array}{ll} \texttt{Binom}(A_{i,j}|D_{i,j},\theta_{0}) & \text{if}\ C_{i,I_{q}}=0 \\ \texttt{Binom}(A_{i,j}|D_{i,j},\theta_{i}) & \text{if}\ C_{i,I_{q}}=1. \end{array} \right. \end{aligned}  $$

We assume beta priors on the *θ* parameters:

$$\begin{array}{@{}rcl@{}} P(\theta_{i}|v_{1}) &=& \texttt{Beta}(\theta_{i}|\alpha_{1},\beta_{1})\\ P(\theta_{0}|v_{0}) &=& \texttt{Beta}(\theta_{0}|\alpha_{0},\beta_{0}),  \end{array} $$

where *v*_1_=(*α*_1_,*β*_1_) and *v*_0_=(*α*_0_,*β*_0_). We denote *v*=(*v*_0_,*v*_1_).

Let *A*_*q*_ be the matrix of alternative allele counts for cells contained in cluster *q*, at mutated positions, i.e., *A*_*q*_=(*A*_*i*,*j*_)_*j*∈*q*,*i*=1,…,*N*_, and let *D*_*q*_=(*D*_*i*,*j*_)_*j*∈*q*,*i*=1,…,*N*_. Since we assume the observed read counts at the different positions and different cells are independent, we have:

$$\begin{array}{@{}rcl@{}} P(A_{q}|D_{q}, I_{q}, \mathbf{C}, \theta, \mathbf{T}) &\,=\,& \prod_{j \in q} \prod_{i=1}^{N} P(A_{i,j}|D_{i,j},I_{q}, C_{i,I_{q}},\theta, T_{j}\,=\,q).  \end{array} $$

### CACTUS model inference

We use Gibbs sampler, a Markov chain Monte Carlo (MCMC) algorithm for generating samples from the posterior distribution. We iteratively sample each hidden variable which is conditionally independent given the rest of the hidden variables in the model. The hidden variables in CACTUS include the cluster assignment matrix **I**, the success probabilities of observing a transcript **θ**=(*θ*_0_,*θ*_1_,…,*θ*_*N*_), the corrected clustering matrix **T**, the corrected genotype matrix **C**, and its error rate *ξ*. We describe the sampling steps for the full joint distribution of these hidden variables in the following.

#### Sampling clone assignment of clusters *I*_*q*_

We sample cluster-to-clone assignment variable *I*_*q*_, given the Markov blanket of *I*_*q*_ in the graphical model (Fig. 2):

1 $$ \begin{aligned} &P(I_{q}=k|\mathbf{A},\mathbf{D},\mathbf{C},\mathbf{T},\mathbf{\theta})\propto P(I_{q}=k)P(A_{q}|D_{q},I_{q} = k, \mathbf{C},\theta,\mathbf{T})\\ &\propto \prod_{j \in q}\prod_{i=1}^{N} \left\{\texttt{Binom}(A_{i,j}|D_{i,j},\theta_{i})^{C_{i,k}} \times \texttt{Binom}(A_{i,j}|D_{i,j},\theta_{0})^{(1-C_{i,k})} \right\}.  \end{aligned}  $$

#### Sampling success probabilities of observing a transcript **θ**

Similarly, we sample *θ* from the posterior probability:

$$\begin{aligned} &P(\theta|\mathbf{A},\mathbf{D},\mathbf{C},\mathbf{I},\mathbf{T},v) \propto P(\theta|v) \prod_{q =1}^{Q}\prod_{j \in q} \prod_{i=1}^{N} P(A_{i,j}|D_{i,j}, I_{q}, C_{i,I_{q}},\theta, T_{j} = q)\\ &\propto \texttt{Beta}(\theta_{0}|\alpha_{0},\beta_{0}) \prod_{i=1}^{N} Beta(\theta_{i}|\alpha_{1},\beta_{1}) \end{aligned} $$

2 $$ \begin{aligned} &\times \prod_{q =1}^{Q}\prod_{j \in q} \prod_{i=1}^{N} \left\{\texttt{Binom}\left(A_{i,j}|D_{i,j},\theta_{i}\right)^{C_{i,I_{q}}} \texttt{Binom}\left(A_{i,j}|D_{i,j},\theta_{0}\right)^{1-C_{i,I_{q}}} \right\}\\ &=\left\{\texttt{Beta}\left(\theta_{0}|\alpha_{0},\beta_{0}\right)\prod_{q =1}^{Q}\prod_{j \in q} \prod_{i=1}^{N}\texttt{Binom}\left(A_{i,j}|D_{i,j},\theta_{0}\right)^{\left(1-C_{i,I_{q}}\right)}\right\} \\ &\times \left\{ \prod_{i=1}^{N} \texttt{Beta}\left(\theta_{i}|\alpha_{1},\beta_{1}\right) \prod_{q=1}^{Q}\prod_{j \in q} \texttt{Binom}\left(A_{i,j}|D_{i,j},\theta_{i}\right)^{C_{i,I_{q}}} \right\}.  \end{aligned}  $$

Using the beta-binomial conjugacy, *θ*_0_ and *θ*_*i*_, for 0<*i*<*N* are sampled from the beta distribution:

3 $$\begin{array}{@{}rcl@{}} &&\theta_{0}|\mathbf{A},\mathbf{C}, \mathbf{I}, \mathbf{T} \sim \texttt{Beta}(\alpha_{0}+u_{0},\beta_{0} + v_{0}),\\ &&\theta_{i}|\mathbf{A},\mathbf{C}, \mathbf{D}, \mathbf{I}, \mathbf{T} \sim \texttt{Beta}(\alpha_{1}+u_{i},\beta_{1} + v_{i}),  \end{array} $$

where

$$\begin{aligned} &u_{0} \!= \sum_{q=1}^{Q}\sum_{j \in q} \sum_{i=1}^{N} A_{i,j} (1\,-\,C_{i,I_{q}}), \quad v_{0} = \sum_{q=1}^{Q}\sum_{j \in q} \sum_{i=1}^{N} (D_{i,j}\,-\,A_{i,j})(1-C_{i,I_{q}}),\\ &u_{i} = \sum_{q=1}^{Q}\sum_{j \in q} A_{i,j}C_{i,I_{q}}, \quad \quad \quad \quad v_{i} = \sum_{q=1}^{Q}\sum_{j \in q} (D_{i,j}-A_{i,j})C_{i,I_{q}}. \end{aligned} $$

#### Sampling the corrected clustering matrix **T**

The corrected sampling matrix **T** is sampled based on the Markov blanket of **T** in the graphical model (Fig. 2):

$$\begin{aligned} P(T_{j}=\ & q|\mathbf{p},\mathbf{A},\mathbf{C},\mathbf{D},\mathbf{I},\theta)\\ =& \frac{P\left(T_{j}=q|p_{j,1},\ldots,p_{j,Q}\right)\prod_{i=1}^{N}P\left(A_{i,j}|D_{i,j}, I_{q}, C_{i,I_{q}}, \theta, T_{j}=q\right)}{\sum_{q^{\prime}=1}^{Q} \!P\!\left(T_{j}\,=\, q^{\prime}|p_{j,1},\ldots\!,p_{j,Q}\right)\! \prod_{i=1}^{N} {\!P\!\left(A_{i,j}|D_{i,j},I_{q},\!C_{i,I_{q^{\prime}}},\theta, T_{j} \,=\, q^{\prime}\!\right)}}, \end{aligned} $$

where we assume the categorical prior over *T*:

4 $$ \begin{aligned} P(T_{j}=&q|\mathbf{p},\mathbf{A},\mathbf{D},\mathbf{C},\mathbf{I},\theta)\\ =& \frac{ p_{j,q} \prod_{i=1}^{N} {P\left(A_{i,j}|D_{i,j},I_{q},C_{i,I_{q}},\theta, T_{j} = q\right)}} { \sum_{q^{\prime}=1}^{Q} p_{j,q^{\prime} }\prod_{i=1}^{N} {P\left(A_{i,j}|D_{i,j},I_{q},C_{i,I_{q^{\prime}}},\theta,T_{j} = q^{\prime} \right)}}.  \end{aligned}  $$

#### Sampling the corrected genotype matrix **C**

Similarly, the corrected genotype matrix **C** is sampled using the Markov blanket of **C** in the graphical model:

5 $$ \begin{aligned} &P(C_{i,k}=1|C_{-(i,k)},\mathbf{A},\mathbf{D},\theta,\mathbf{I},\xi,\Omega_{i,k},\mathbf{T}) =\\ &\frac{ {|\Omega_{i,k}-\xi|}\prod\limits_{q=1}^{Q}\prod\limits_{j\in q} \texttt{Binom}(A_{i,j}|D_{i,j},\theta_{i})^{\mathbbm{1}_{(I_{q}=k)}}}{|\Omega_{i,k}\,-\,\xi|\!\prod\limits_{q=1}^{Q}\prod\limits_{j\in q} \!\texttt{Binom}(A_{i,j}|D_{i,j},\theta_{i})^{\mathbbm{1}_{(I_{q}=k)}} \!+ \!(1\,-\,|\Omega_{i,k\!}-\!\xi|)\prod\limits_{q=1}^{Q}\prod\limits_{j\in q} \texttt{\!Binom}(A_{i,j}|D_{i,j},\theta_{0})^{\mathbbm{1}_{(I_{q}=k)}}},\\  \end{aligned}  $$

where

$$\begin{aligned} |\Omega_{i,k}&-\xi|\prod\limits_{q=1}^{Q}\prod\limits_{j\in q} \texttt{Binom}(A_{i,j}|D_{i,j},\theta_{i})^{\mathbbm{1}_{(I_{q}=k)}}\\ =& P(C_{i,k}=1|\Omega_{i,k},\xi)\prod\limits_{q=1}^{Q}\prod\limits_{j\in q} P(A_{i,j}|D_{i,j},I_{q},C_{i,I_{q}}=1,\theta, T_{j} = q) \end{aligned} $$

and

$$\begin{aligned} (1&-|\Omega_{i,k}-\xi|)\prod\limits_{q=1}^{Q}\prod\limits_{j\in q} \texttt{Binom}(A_{i,j}|D_{i,j},\theta_{0})^{\mathbbm{1}_{(I_{q}=k)}} \\=& P(C_{i,k}=0|\Omega_{i,k},\xi)\prod\limits_{q=1}^{Q}\prod\limits_{j\in q} P(A_{i,j}|D_{i,j},I_{q},C_{i,I_{q}}=0,\theta, T_{j} = q). \end{aligned} $$

Here, we assume Bernoulli distribution over **C**_*i*,*k*_:

$$P(C_{i,k}=1|\Omega_{i,k},\xi) = \xi^{1-\Omega_{i,k}} \times (1-\xi)^{\Omega_{i,k}}$$

Indeed, we have *P*(*C*_*i*,*k*_=1|*Ω*_*i*,*k*_=1,*ξ*)=1−*ξ* and *P*(*C*_*i*,*k*_=1|*Ω*_*i*,*k*_=0,*ξ*)=*ξ*. Thus, we can shortly write *P*(*C*_*i*,*k*_=1|*Ω*_*i*,*k*_,*ξ*)=|*Ω*_*i*,*k*_−*ξ*|. Similarly, for *C*_*i*,*k*_=0, we can write *P*(*C*_*i*,*k*_=0|*Ω*_*i*,*k*_,*ξ*)=1−|*Ω*_*i*,*k*_−*ξ*|.

#### Sampling the error rate *ξ*

We can compute the distribution of the error rate *ξ* having the corrected genotype matrix *C*, as well as the input clone genotype matrix *Ω* and hyperparameters *κ* as follows:

$$\begin{array}{@{}rcl@{}} P(\xi|C,\Omega,\kappa) = P(\xi | \kappa)\prod_{i}^{N}\prod_{k}^{K} P(C_{i,k}=1|\Omega_{i,k},\xi)\\ = \texttt{Beta}(\xi;\kappa_{0},\kappa_{1}) \times \xi^{1-\Omega_{i,k}} (1-\xi)^{\Omega_{i,k}}. \end{array} $$

From the beta-Bernoulli conjugacy we obtain:

6 $$ \begin{aligned} P(\xi|\mathbf{C},\Omega,\kappa)\! = \!\texttt{Beta} \left(\kappa_{0} \,+\, \sum_{i,k}\mathbbm{1}(\Omega_{i,k} \neq C_{i,k}),\kappa_{1} \,+\, \sum_{i,k}\mathbbm{1}(\Omega_{i,k} \,=\, C_{i,k}) \right).  \end{aligned}  $$

Finally, the Gibbs sampling algorithm for CACTUS was derived as a straightforward modification of the algorithm used for cardelino [21]. In the algorithm, *I*_*q*_ is iteratively sampled using Eq. (1) for *q*=1,…*Q*,*θ*_*i*_ for *i*=1,…,*N* is sampled using Eq. (3), *T*_*j*_ is sampled for *j*=1,…,*M* using Eq. (4), *C*_*i*,*k*_ for *i*=1,…,*N* and *k*=1,…*K* is sampled using Eq. (5), and *ξ* is sampled using Eq. (6).

## Results

### Single cell and WES profiling of two FL patients

The analyzed tumor cell populations were collected from lymph nodes of two FL patients: a male patient (S144) at the age of 37, who was diagnosed with an IgM expressing FL stage IV and a female patient (S12118) at the age of 51, who was diagnosed with an IgG expressing FL stage IV. To detect (sub-)clonal mutations, we performed WES at 200 × coverage and called mutations between FL cells and paired stromal non-hematopoietic cells. We detected 398 somatic mutations for patient S144 and 1034 somatic mutations for patient S12118 with somatic *p* value (SPV) <0.1.

Next, for pooled samples of both subjects, we performed single-cell sequencing of purified FL cells for full transcriptomes and BCR enriched libraries. We used the Vireo method [29] to group single cells back to the patients based on matching of alleles expressed in the single cells with germline mutations detected by bulk WES. Deconvolution of the whole transcriptome data yielded 1524 cells of subject S144 and 874 cells of subject S12118, respectively. BCR sequencing yielded BCR heavy chain sequences for approx. 70% of cells in both patients. Both samples were dominated by a limited number of larger BCR clusters (further referred to as multiplet BCR clusters), with many BCR clusters containing only one element (singleton BCR clusters). The “Pielou evenness index” was 0.59 for S144 and 0.53 for S12118, indicating moderate intraclonal diversification [30]. For generality, cells without BCR heavy chain sequences were considered to form a separate singleton cluster (see Additional file 1 for BCR cluster size distribution).

### A probabilistic model for assigning cell clusters to evolutionary tumor clones.

CACTUS is a Bayesian method that integrates three different sources of prior knowledge: (1) a set of tumor clones with their genotypes, (2) independently obtained non-overlapping cell clusters, and (3) scRNA-seq transcripts at mutated sites, to map each cell cluster to its corresponding tumor clone (the “Methods” section). Cells of the same cluster are assumed to come from the same tumor clone. Since the clusters are non-overlapping sets of cells, the cluster assignment to clones defines also the cell assignment (each cell in a given cluster is assigned to the same clone as its cluster).

Here, the input cell clustering was defined by the BCR sequences. Cells with the same BCR sequence are expected to be more likely to come from the same tumor clone. Thus, here CACTUS takes advantage of the extra information of BCR sequences to gain power and confidence of the assignment. During model inference, both the input clone genotypes and the input cell clustering are corrected, taking into account all available data. Thus, although the input clusters are defined as sets of cells with identical BCR sequences, during model inference, the cells may swap between clusters, based not only on BCR sequence similarity but also based on shared sets of mutations.

CACTUS yields the posterior probability estimate for each given cell cluster to be mapped to each given clone. This probability is defined using a beta-binomial model for the allele-specific transcript counts for each mutation and cell in this cluster. The model estimates the error rate for the given imperfect genotypes of the clones and outputs corrected genotypes. Similarly, the corrected clustering of single cells is returned. The likelihood of assigning a cluster to a given clone increases with the similarity of the mutation signal observed in the cells of the corrected cluster to the corrected genotype of that clone. Overall, the three most important hidden variables in the model are the corrected clone genotypes, the corrected clusters, and the assignment of corrected clusters to the clones by matching to their corrected genotypes. The final assignment of the clusters (and thus also their contained single cells) is obtained by selecting the most probable tumor clone for each corrected cluster (Fig. 1).

For both subjects, to define the input clonal structures, we first identified a set of mutations that could be identified both in WES and scRNA-seq data. We consider the mutation to be present in scRNA-seq if at least one variant read is observed. From the identified 398 mutations with SPV <0.1 for subject S144 and 1034 mutations for subject S12118, for further analysis, we selected only these mutations, for which any transcript expression was observed in scRNA-seq. Despite the relaxed significance level of 0.1 for the somatic *p* values, we consider the common mutations as reliable, since they have evidence in both data sources. Only 5 out of 95 total resulting common mutations for subject S144, and 5 out of 133 common mutations for subject S12118, had somatic *p* value in the (0.05,0.1) interval (Additional file 1). Numbers of the common mutations vary in different cells (Additional file 1). For further analysis, we considered only cells which contain at least one of the common mutations. This included 1262 out of 1524 cells in subject S144 and 799 out of 874 cells in subject S12118.

We next applied Canopy to the WES data for the common mutations and extracted the top tree and its corresponding clones, with their genotypes. To obtain the cell-to-clone assignment, CACTUS was applied to the obtained clonal structure, with a clustering of single cells defined by identical BCR sequences and scRNA-seq transcript counts as input. To demonstrate how the addition of the BCR clustering information improves the assignment of cells to clones, we applied cardelino [21] to the same Canopy trees and the scRNA-seq transcript counts. From these data, cardelino derived cell assignment to tumor clones. The two models (CACTUS and cardelino) are similar, but CACTUS can exploit the data more fully as it additionally takes into account the cell clustering (here, by BCR sequence) information into account. In fact, for the specific case of such uninformative clusters that contain exactly one cell, CACTUS reduces to cardelino. Thus, naturally, the advantage of CACTUS should be visible for such cells that are contained in clusters of more than one cell. It is important to note that both CACTUS and cardelino correct the input clone genotypes in their own way. Thus, the final genotypes of the clones might be similar, but obtained by correcting different initial clone genotypes. Therefore, keeping original labels of the clones would introduce artificial differences between the outputs of the two methods. To make a comparison of CACTUS to cardelino feasible, we first adjust the clone labels in such a way that clones with most similar corrected genotypes between the two methods share the same label (Additional file 1).

### CACTUS solution verified by an independent gene expression analysis

**Fig. 3:**
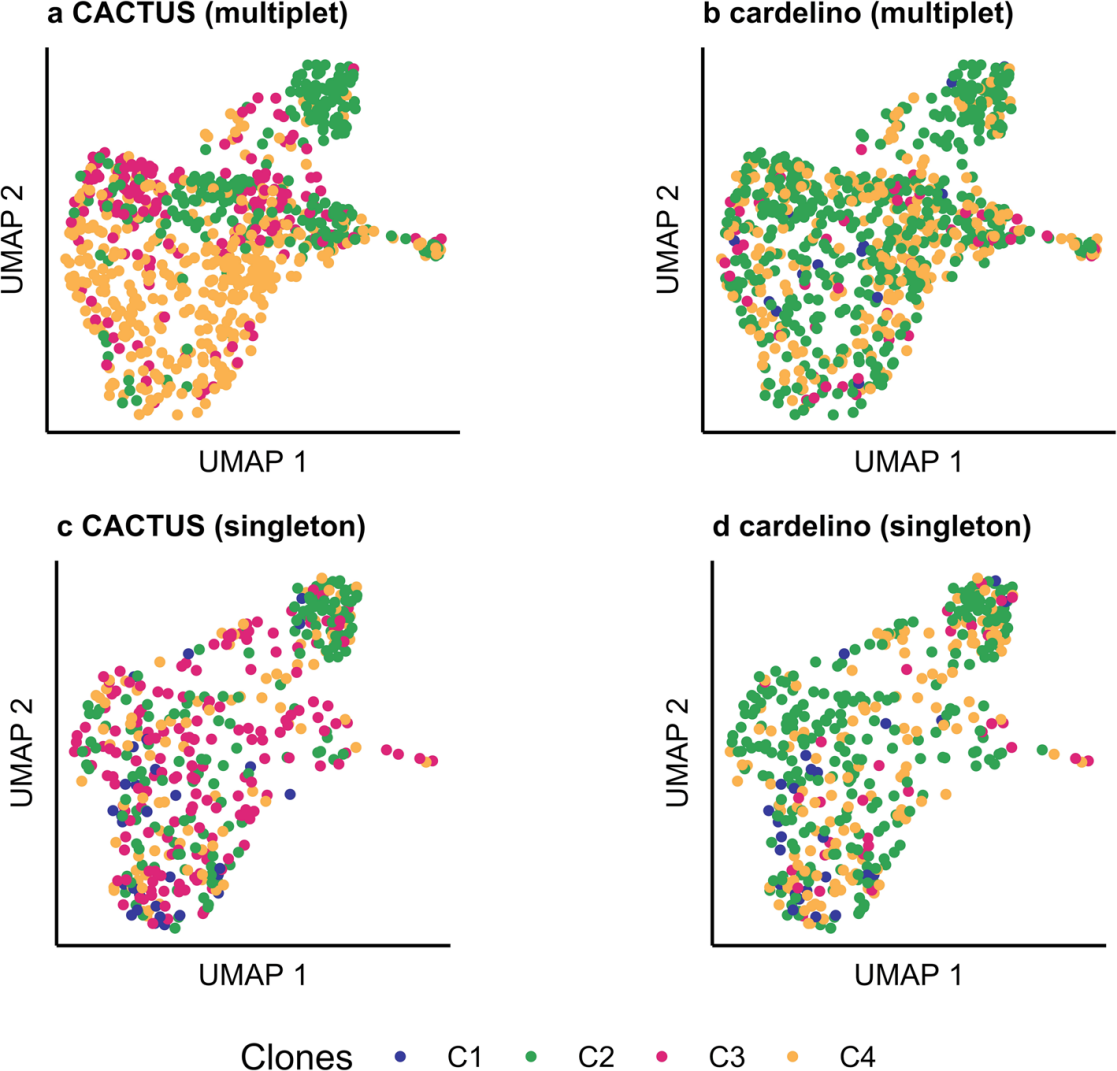
Validation of cell-to-clone assignment with gene expression for subject S144. **a, b, c, d** Transcript expression of the cells reduced to two dimensions using UMAP, shown separately for the cells in multiplet BCR clusters (**a**, **b**) and for cells belonging to singleton BCR clusters (**c,d**). Each point corresponding to a cell is colored by its clone assigned by CACTUS (**a**, **c**) and by cardelino [21] (**b**, **d**). The advantage of CACTUS in terms of agreement with gene expression is more pronounced for cells in multiplet BCR clusters

**Fig. 4:**
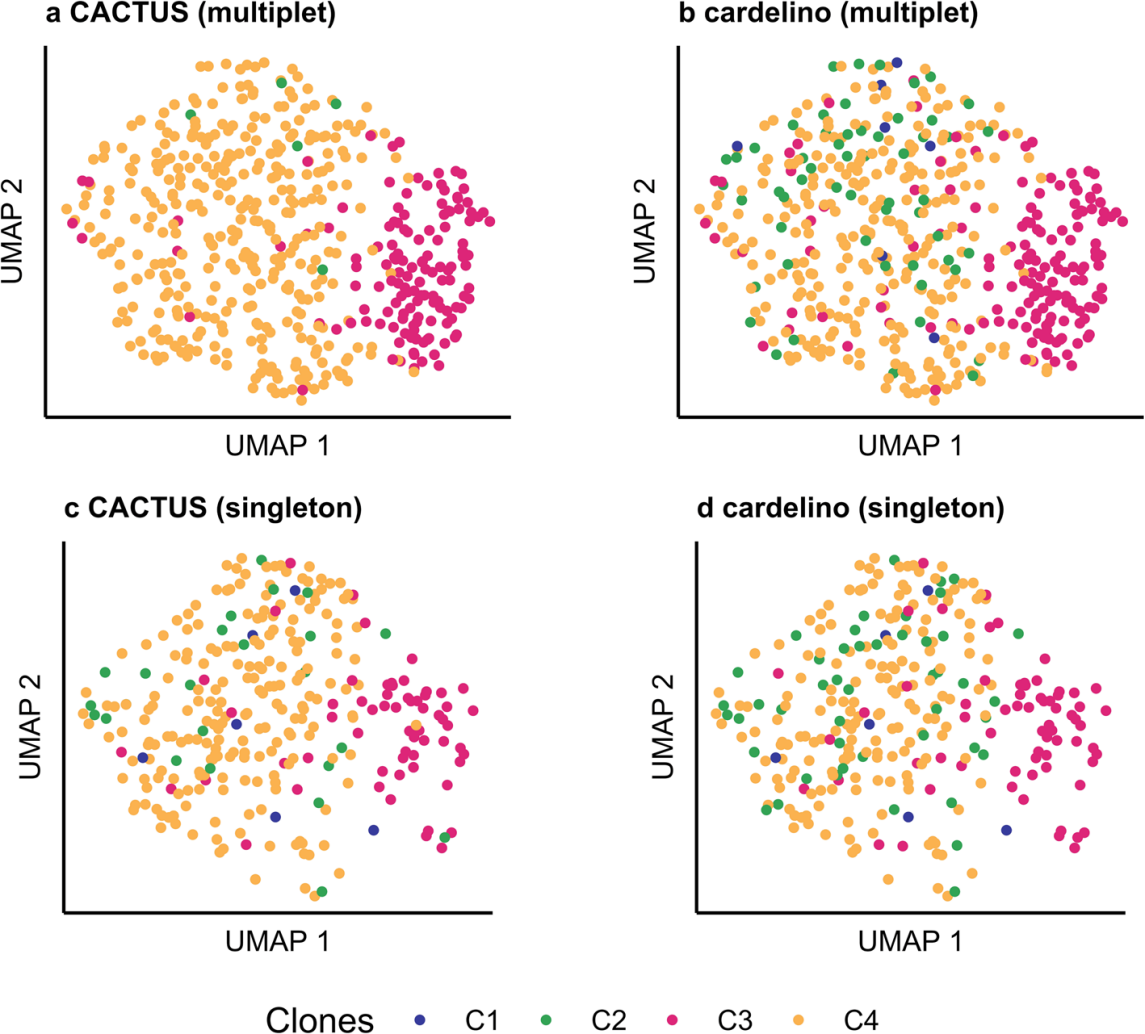
Validation of cell-to-clone assignment with gene expression for subject S12118. Figure panels as for subject S144 in Fig. 3. Also for subject S12118, assignment to clones for cells in multiplet BCR clusters using CACTUS (**a**) improves agreement with gene expression data compared to assignment of cells in singleton BCR clusters (**d**) and assignment using cardelino [21] (**b**), as quantified using connectivity measure (**c**). For singleton BCR clusters, CACTUS performs comparably well as cardelino

To validate the returned cluster-to-clone assignment and the induced cell assignment, we performed independent analysis of transcript expression levels obtained from scRNA-seq of the same cells. Note that here, we describe gene expression as independent data since the transcript counts across all sites in the gene sequences are not used by CACTUS during inference. In contrast, CACTUS uses specific counts of those reads that map to the variant sites. Gene expression information is thus not used for model inference, only the signal for existence of mutations. We investigated whether the grouping of cells into the inferred clones tends to coincide with similarity of their expression profiles visually (Figs. 3 and 4). To this end, we reduced the dimensionality of expression data using UMAP [31] provided in the Seurat package [32] and colored each cell with its corresponding clone inferred using CACTUS, and for a comparison, cardelino [21].

As expected, CACTUS leverages information obtained from the multiplet BCR clusters. For cells in such BCR clusters, the results of CACTUS are more consistent with gene expression (visualized for UMAP in Figs. 3a and 4a) than the results of cardelino (Figs. 3b and 4b). For subject S144 and cells contained in the multiplet BCR clusters, CACTUS identifies clone C2 as a set of cells that is separated in gene expression space from a large cluster of cells, which is populated mostly by clone C4 and in part by clone C3. In contrast, cardelino finds clones which are mixed in the reduced gene expression space (Fig. 3a,b). For subject S12118, both methods associate clone C3 with one gene expression cluster and clone C4 with another, with the two gene expression clusters clearly separated in the reduced space. For CACTUS, the identified clones are slightly less intermixed with others than for cardelino (Fig. 4). For CACTUS, the clone assignments of cells in the singleton BCR clusters show less agreement with expression than assignments of cells in multiplet clusters (Figs. 3c and 4c). The agreement for those cells is comparably low for cardelino (Figs. 3d and 4d).

To quantify the agreement of the obtained assignment of cells to the clones with gene expression, we used several quality measures [33]. To this end, for each cell and each subject, we first reduced the dimension of the normalised expression measurement to 25 using PCA. Next, we computed the root mean square standard deviation (RMSSTD), connectivity, Dunn index, and Calinski-Harabasz (CH) index for the reduced gene expression vectors, grouped according to the assignment of cells to the clones [34–38] (Table 1). In this way, we measured to what extent the gene expression of the cells inside each clone is homogeneous and differs between the clones. A RMSSTD is a measure of compactness—a low value of RMSSTD indicates low variance of gene expression in each set of cells assigned to the same clone. The connectivity measure takes values between 0 and infinity and uses the *k*-nearest neighbors to indicate the degree of connectedness of the clusters. We used *k*=10 for the computation, but we noted that other values of *k* gave similar results. If the cells assigned to the same clone would also be close in terms of Euclidean distance in the reduced 25-dimensional expression space, the connectivity would be minimized. High Dunn index values imply increased compactness of each clone and better separation between the clones, computed for the reduced expression profiles of cells assigned to the clones. The CH index is another measure for evaluating both compactness and separation simultaneously, using average between and within clone sum of squares. The higher CH score indicates more agreement of the assignment of cells into clones with their gene expression values. For cells in the multiplet BCR clusters, these quality measures clearly indicate that CACTUS obtains better agreement between cell-to-clone assignment and gene expression than cardelino (Table 1). In contrast, for cells in singleton clusters, CACTUS obtains similar quality measures as cardelino.

**Table 1:** Quantification of the agreement of the cell-to-clone assignment with gene expression profiles of the cells

Subject	Type	Method	Dunn index	RMSSTD	CH	Connectivity
S144	Multiplet	CACTUS	**0.066**	**57.0**	**15.6**	**898.9**
		cardelino	0.057	77.1	3.2	1250.9
	Singleton	CACTUS	**0.054**	110.9	**3.2**	839.5
		cardelino	0.052	**109.5**	1.9	**711.9**
S12118	Multiplet	CACTUS	**0.098**	**79.2**	**11.9**	**169.6**
		cardelino	0.084	96.2	10.0	495.0
	Singleton	CACTUS	0.085	105.4	**4.1**	**285.4**
		cardelino	**0.092**	**99.4**	3.9	396.5

Bolded values indicate which method (CACTUS or cardelino) obtained better agreement for the given subject and type of cluster that the cells assigned to the clones come from. High values of the Dunn index and the Calinski-Harabasz (CH) index, as well as low values of the root mean square standard deviation (RMSSTD) and connectivity quantify to what extent the gene expression of the cells is homogeneous inside each clone and differs between the clones

We performed independent clustering of cells by their normalised expression using Seurat [32]. Then, we compared the resulting clustering of cells by expression to the grouping of cells to clones inferred by CACTUS and by cardelino using the adjusted Rand index (ARI; [39]). The index, with values in the [ − 1,1] interval, is a corrected-for-chance version of the Rand index, measuring similarity between two given clusterings. ARI is negative when the agreement is lower than expected by chance and is maximized when the compared clusterings are identical. For subject S144 and the cells that are in the singleton BCR clusters, both clones inferred by CACTUS and by cardelino show very low similarity to expression clusters (with ARI 0.03 and 0.02, respectively). Compared to cardelino (ARI 0.01), CACTUS achieves a higher agreement with the gene expression clustering for cells contained in the multiplet BCR clusters (ARI 0.13). For subject S12118, the CACTUS clones have the same similarity to expression clusters as cardelino. For cells that are in the singleton BCR clusters, both CACTUS and cardelino yield ARI of 0.12. Finally, for the cells in the multiplet BCR clusters, the ARI for both CACTUS and cardelino is 0.21.

Overall, these results indicate that by accounting for the BCR sequence similarity, CACTUS improves the genotype-to-gene expression phenotype mapping.

### CACTUS enhances the confidence of cell-to-clone assignment

**Fig. 5:**
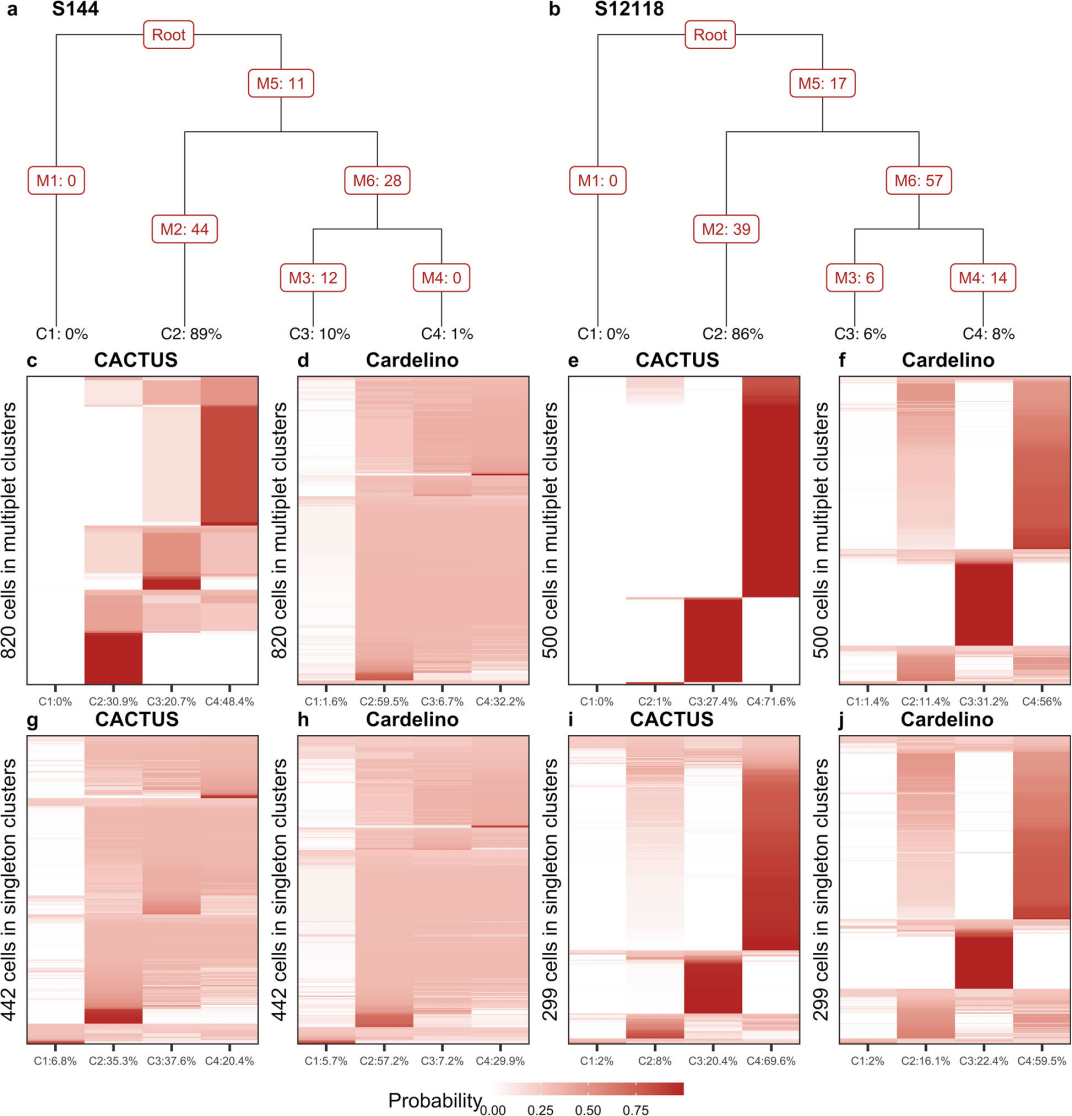
Confidence of cell assignment to the tumor clones. **a, b** Evolutionary trees inferred by Canopy [9] for subject S144 (**a**) and S12118 (**b**). Leaf labels: clone prevalences. Branch labels: numbers of acquired mutations. Canopy considers also CNVs, but they are not used for cell-to-clone mapping and hence not visualized here. Thus, the branch labels can be zero when the alterations acquired along that branch are copy number changes. Clone 1 corresponds to the base, normal clone. In tree **a**, clone 4 (C4) differs from clone 3 (C3) by the 12 SNVs acquired on the branch leading to the leaf C3. **c–j** Shades of brown indicate the probability of assignment of cells (y axis) to the clones (x axis; labeled with corrected prevalences, computed as the fraction of single cells assigned to the clones) by CACTUS (**c, g, e, i**) and cardelino [21] (**d, h, f, j**). For cells in multiplet BCR clusters (second row), CACTUS yields higher confidence of cell-to-clone assignment (**c, e**) than cardelino (**d, f**). For cells in singleton BCR clusters (third row) for subject S144, the confidence of cell-to-clone assignment by CACTUS (**g**) is similarly weak as by cardelino (**h**), while for S12118 and for CACTUS (**i**), the confidence is higher than for cardelino (**j**)

[For both subjects, the top identified evolutionary trees consisted of four clones (Fig. 5a, b). The number of mutations acquired along the branches of the trees ranges from 0 to 57. The genotype of each input clone is defined as the set of the mutations acquired on the path from the root of the tree to the leaf corresponding to the clone (Additional file 2). Notably, the clone genotypes and frequencies derived by Canopy (Fig. 5a, b) were corrected both by CACTUS (Fig. 5c, g, e, i) and cardelino (Fig. 5d, h, f, j). CACTUS, in addition, corrected the input BCR clustering. All results discussed below are for the corrected genotypes and corrected clusters.

We investigated the confidence of assignment of cells to the tumor clones for both subjects (Fig. 5). The assignment of cells to the clones was directly derived from the assignment of their BCR clusters. In general, thanks to the additional information from the BCR clusters, CACTUS assigns cells to clones with a clearly higher confidence than cardelino [21]. From both methods, the probability of assigning each cell to each clone can be derived as output. For subject S144 and a majority of cells, the probability of assignment by cardelino is almost uniform across the clones (Fig. 5d, h). In contrast, for the subset of cells in the multiplet BCR clusters, the probability of assignment by CACTUS makes confident assignments (Fig. 5c). For the cells in the singleton BCR clusters, CACTUS assigns cells with similar confidence to cardelino (Fig. 5g).

Compared to S144, for subject S12118 the confidence of assignment is larger for both methods (Fig. 5). Again, CACTUS has an advantage over cardelino, especially for cells in the multiplet BCR clusters, assigning majority of those cells to one clone with high probability (Fig. 5e, i). In contrast, for a majority of cells, cardelino yields similar probabilities of assignment to clones C2 and C4 (Fig. 5f, j).

Overall, the confidence of the assignment is clearly higher for CACTUS than for cardelino, for both subjects (Table 2). Here, we quantified confidence as the concentration of the assignment probability distribution over the clones, averaged over the cells, using the measures of entropy and the Gini index [40, 41]. Both entropy and Gini index should be lower for larger concentration of the probability distribution (equivalently, smaller dispersion).

**Table 2:** Quantification of the confidence of cell-to-clone assignment

Subject	Type	Method	Entropy	Gini index
S144	Multiplet	CACTUS	**0.42**	**0.46**
		cardelino	0.85	0.90
	Singleton	CACTUS	**0.79**	**0.84**
		cardelino	0.87	0.90
S12118	Multiplet	CACTUS	**0.04**	**0.04**
		cardelino	0.39	0.45
	Singleton	CACTUS	**0.36**	**0.38**
		cardelino	0.47	0.54

Confidence is measured as the concentration of the probability distribution of assigning a cell to clones, averaged across cells. Bolded values indicate which method (CACTUS or cardelino) obtained higher confidence. Both normalized entropy (entropy divided by the maximum possible value) and the Gini index are supposed to have lower values for more concentrated distributions, and larger values for more dispersed ones

### Assignment of BCR clusters to tumor clones

**Fig. 6:**
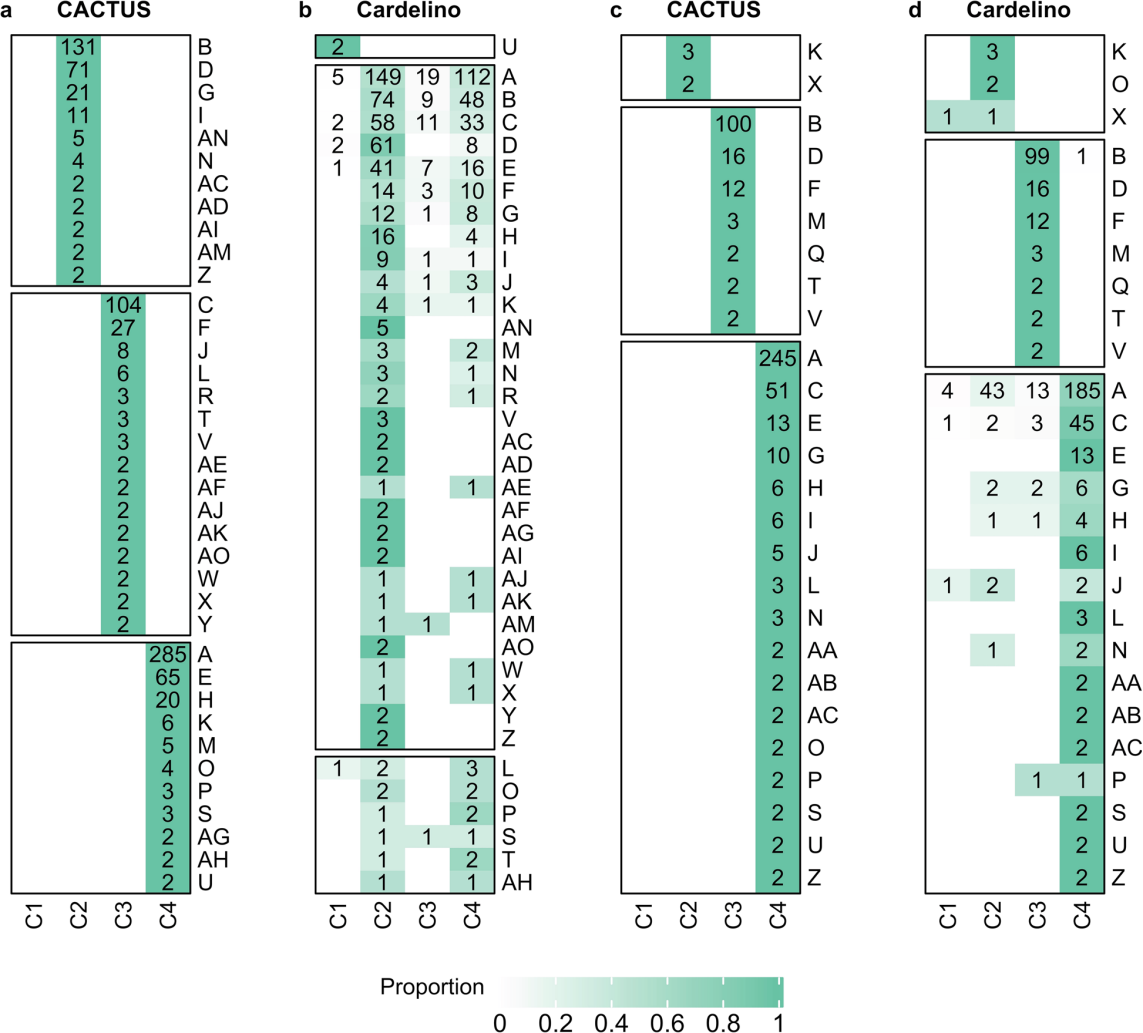
BCR cluster assignment to tumor clones, for both subjects. S144 (**a, b**) and S12118 (**c, d**), using CACTUS (**a, c**) and cardelino [21] (**b, d**). Heatmaps with shades of green indicate the proportion of cells in multiplet cluster (y axis) assigned to clones (x axis). Each number in a green entry indicates the non-zero number of cells of the corresponding BCR clusters assigned to the corresponding clone. Only BCR clusters of at least two cells are featured. As expected, for both subjects, CACTUS assigns entire BCR clusters to single clones (**a, c**). For cardelino, the proportions of BCR clusters are more distributed across the clones (**b, d**)

Finally, we inspected the assignment of BCR clusters to clones by CACTUS. For a comparison, for each clone, we computed the proportion of each multiplet BCR cluster (the fraction of cells in that BCR cluster) that were assigned to this clone using cardelino (Fig. 6). In the case of ties in the highest proportions across clones, we assumed the BCR cluster was assigned to the same clone as by CACTUS.

As expected by construction of the underlying probabilistic model, for both subjects, CACTUS assigns entire BCR clusters to single clones (Fig. 6a, c). For cardelino, the proportions of BCR clusters are more distributed across the clones (Fig. 6b, d). Given the uncertainty of assignment of cells to clones by cardelino for subject S144 (Fig. 5), it is not surprising that for some of the BCR clusters, the clone assigned by CACTUS does not agree with the clone with the highest proportion of cells assigned by cardelino. CACTUS did not assign any BCR cluster to clone C1, while cardelino assigned cluster U to that clone. All of 11 BCR clusters assigned to clone C2 by CACTUS were assigned to the same clone by cardelino. Out of 15 BCR clusters assigned to clone C3 by CACTUS, however, none was assigned to clone C3 also by cardelino. This large disagreement comes mainly from the fact that cardelino assigned the highest proportion of cells contained in 13 of these 15 clusters again to clone C2. Finally, out of 11 BCR clusters assigned to clone C4 by CACTUS, 4 were assigned in the highest proportion to the same clone also by cardelino.

For subject S12118, the assignment of cluster agrees between the two methods, with the only exception of cluster O. This is in accordance with the increased confidence of assignment of cells to clones by both methods for that subject (compare Fig. 5).

In summary, the agreement of both cell-to-clone and BCR cluster-to-clone mapping between the CACTUS and cardelino increases with the confidence of assignment. For subject S144, for which cardelino yielded low-confidence assignments, 736 out of 1262 cells in total (58%) and 22 out of 37 multiplet BCR clusters (59%) were assigned to different clones by the two methods. Here, we assume cardelino assigns a BCR cluster to the clone to which it assigned the highest proportion of cells. For subject S12118, where both methods increased confidence of assignment, only 123 cells out of 799 (15%) and only one BCR cluster out of 26 multiplet BCR clusters (4%) was assigned differently.

## Discussion

Here, we propose a probabilistic model for accurate and confident mapping of single tumor cells to their evolutionary clones of origin. In this way, it allows clone-specific gene expression profiling, opening the possibility to reconstruct genotype-to-phenotype maps. The task of cell-to-clone mapping is challenged by multiple technical obstacles. First, although multiple methods exist for the inference of tumor evolution, resolving tumor clones and their genotypes is in itself a difficult computational problem and errors are expected [12]. Thus, CACTUS uses the additional signal both in the scRNA-seq and in clustering data to correct the given genotypes of the clones. Second, the information in scRNA-seq data is only sparse, prone to errors such as dropout and uneven coverage, and biased to mutations observable in typically sequenced first 150 nt of transcripts. It is thus important to realize that the analysed tumor history is limited only to the mutations measurable in single cells and is potentially more coarse-grained than the true clonal structure of the tumor. These limitations are purely technical, and in this respect analysis using CACTUS would benefit from full-length transcript sequencing with high depth, as well as further developments increasing the quality of scRNA-seq technology.

The key aspect of our model is the ability to borrow information across different measurements (both of DNA and RNA) of the cells in the sample. In particular, in addition to clone genotypes derived from WES, and allele-specific transcript counts measured using scRNA-seq, the model leverages information given by independent clustering of single cells. Our results show that this additional evidence is crucial to overcome the challenges of the cell-to-clone assignment problem. Not any given cell clustering, however, can empower CACTUS to deliver more confident results. The assumption that cells contained in the same cluster tend to belong to the same clone is critical for model performance. In particular, such cell clustering, where the cells in the same cluster are not expected to belong to the same clone, can misguide model inference. Apart from clustering by genomic features, which is expected to agree with the clonal structure of the tumor cell population, for example, clustering by location in the tissue could be provided as input to CACTUS. Here, we used single-cell BCR heavy chain sequences to define the input clustering. As would other relevant genomic features, mutations in BCR loci bring evolutionary information. On a general level, they indicate whether a subpopulation of tumor cells sharing a BCR sequence with a low number of BCR mutations evolved relatively early, or if it has more recently evolved and carries a higher number of mutations. Similar BCR sequences indicate common evolutionary origin, as otherwise they would be disrupted by acquisition of additional mutations. Importantly, although the input clustering is defined by identical BCR sequences, cells are shifted between clusters during the model inference process, both re-distributing cells among multiplet clusters and joining singleton clusters to multiplets. This process is influenced by all available data, i.e., not only the similarity of BCR sequences, but also the variants found in scRNA-seq and in the genotypes derived from WES. Here, the quality of additional information brought in by the BCR clusters is assured by the complete and deep sequencing coverage of BCR loci in the applied scRNA-seq strategy. Errors in sequencing, however, may still occur, which further supports the need for updating the input cell clusters.

CACTUS could be extended in the future to further broaden its functionality and to account for even more additional measurements. The input clone genotypes and the number of clones are corrected, but need to be inferred a priori to applying the model, and the evolutionary tree structure is not utilized by the model. The possible errors in the prior tree inference, or a wrong assumption about the number of clones, can potentially hamper the model performance. To some extent, this problem is avoided by the fact that CACTUS corrects the input clone genotypes during inference. Instead, CACTUS could be extended to simultaneously infer the evolutionary tree, yielding the clones and their genotypes, together with the cell assignment to the clones. Finally, other measurements could be incorporated to statistically strengthen model inference. For example, gene expression similarities between cells, here used for model validation, could be used as input, as cells with similar expression profiles are expected to come from the same clone.

The model is applied to newly generated FL patient data, for the first time shedding light on how clonal evolution in this cancer type induces clone-specific gene expression and agrees with BCR clusters. Accurate mapping of clonal structures with gene expression patterns allows detection of potential therapy-resistant clones, which is essential for effective personalized treatment. Our results demonstrate applicability of CACTUS to the complex cancer samples. The model, however, is more generally applicable and can describe somatic evolution also in other diseases or in the healthy tissue.

## Conclusions

Here, we deal with the task of gene expression profiling of tumor clones by matching the genotypes of the clones to the mutations found by RNA sequencing in the single cells. As applied here, CACTUS benefits from the additional information contained in clusters of single cells sharing similar BCR sequences to assign cells to clones, to successfully deal with errors and dropouts in single-cell RNA sequencing, and the difficulty of inferring the correct clonal structure. In summary, this contribution is a step forward in establishing computational tools for resolving the tumor heterogeneity and, by combining genotype with gene expression profiles, its impact on functional diversification of the tumor cell subpopulations.

## Supplementary Information

**Additional file 1** Figures S1-S6

**Additional file 2** Table S1

## Data Availability

CACTUS is freely available as an R code at our GitHub repository [42]. The results were plotted using *ggplot2* and *ComplexHeatmap* packages[43, 44]. The dataset supporting the conclusions of this article is available at our GitHub repository [45].
