# Supplementary material for: CACTUS: integrating clonal architecture with genomic clustering and transcriptome profiling of single tumor cells
Source: Genome Med. 2021 Mar 24;13:45. doi: 10.1186/s13073-021-00842-w (PMC7988935; doi:10.1186/s13073-021-00842-w)
Supplement: Supplementary file 1 — Additional file 1 Figures S1-S6 [file 13073_2021_842_MOESM1_ESM.pdf]

CACTUS: integrating clonal architecture with genomic clustering and transcriptome profiling of single tumor cells

Supplementary Figures

Shadi Darvish Shafighi[1†], Szymon M. Kiełbasa[2†] , Julieta Sepulveda-Yanez[3], Ramin Monajemi[2], Davy Cats[2], Hailiang Mei[2], Roberta Menafra[4], Susan Kloet[4], Hendrik Veelken[3], Cornelis A.M. van Bergen[3§], Ewa Szczurek[1§*]

[1]Faculty of Mathematics, Informatics, and Mechanics, University of Warsaw, Poland,
[2]Department of Biomedical Data Sciences, Leiden University Medical Center, The Netherlands,
[3]Department of Hematology, Leiden University Medical Center, The Netherlands,
[4]Leiden Genome Technology Center, Leiden University Medical Center, The Netherlands.

[†,§]Equal contribution. *To whom correspondence should be addressed.

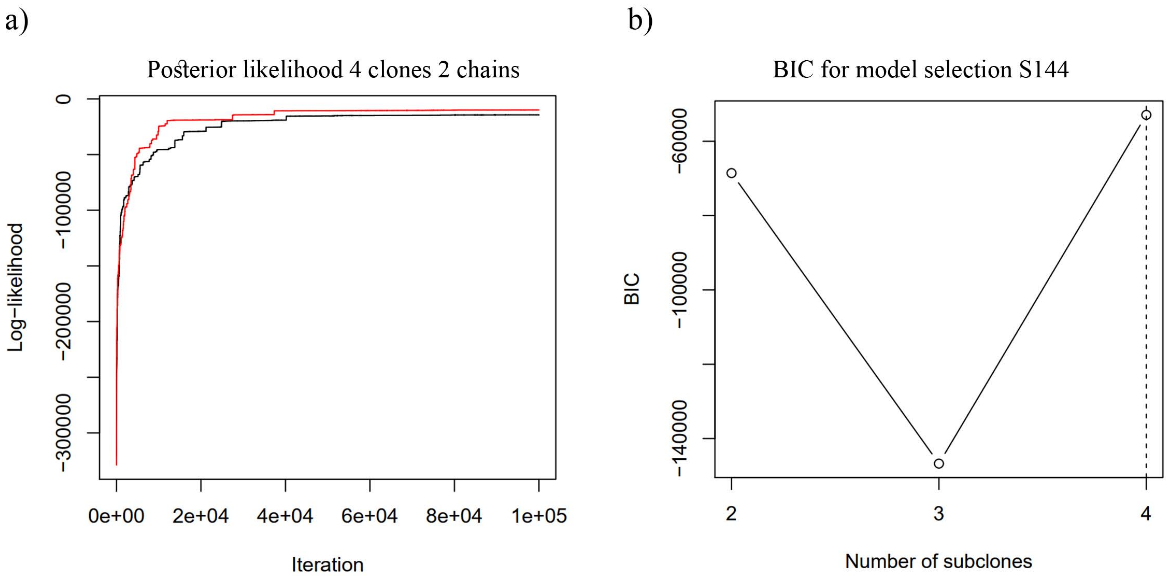

Fig S1: **Posterior likelihood (a) and BIC (b) obtained by the Canopy model** for subject S144. Canopy infers tumor evolution trees using MCMC. **a** "Posterior likelihood" plot, where the log-likelihood is plotted against the iteration number for two chains. Inspection of this plot, as recommended in the Canopy vignette, indicates that the sampling has converged. **b** The BIC criterion allows for a comparison between models with different numbers of parameters. Here, the BIC (y-axis) for tree sizes from 2 to 4 clones; (x-axis) indicates that the tree with four clones is the most likely model of the evolution of the tumor of subjectt S144.

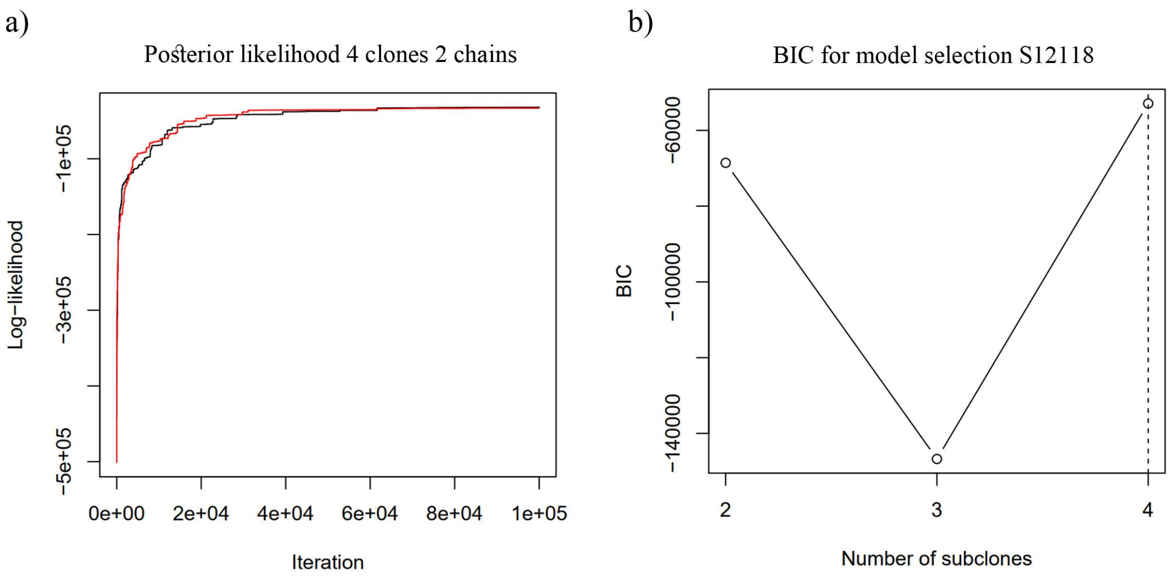

Fig S2: **Posterior (a) and BIC (b) obtained by the Canopy model** for subject S12118. Plot axes as in Figure S5. **a** Inspection of the "Posterior likelihood" plot for subject S12118 indicates that the sampling has converged. **b** BIC indicates that from the three considered options of the number of clones (from 2 to 4) the tree with four clones is the most likely model of the evolution of the tumor of subject S12118.

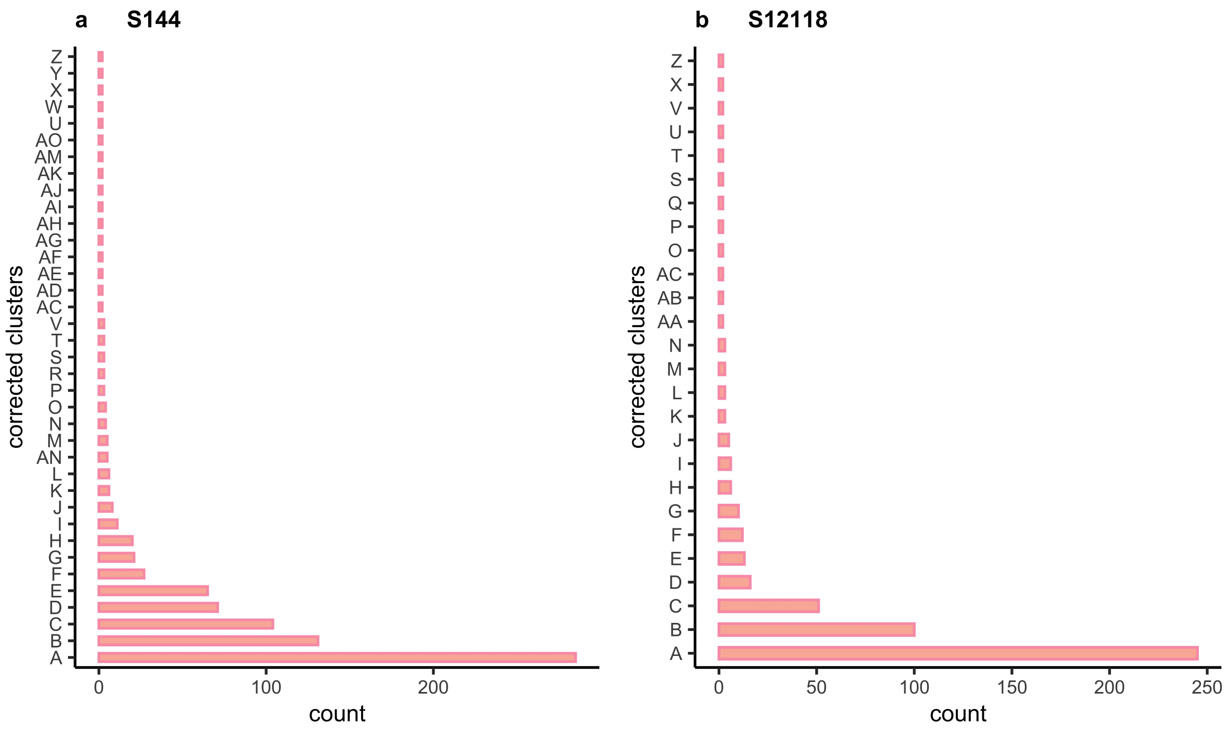

Fig S3: **Number of cells belonging to each input BCR cluster** for (a) subject S144 and (b) subject S12118. Only numbers of cells in multiplet clusters are plotted. For the input clustering, there are 442 singleton clusters for subject S144 and 299 single cell clusters subject S12118.

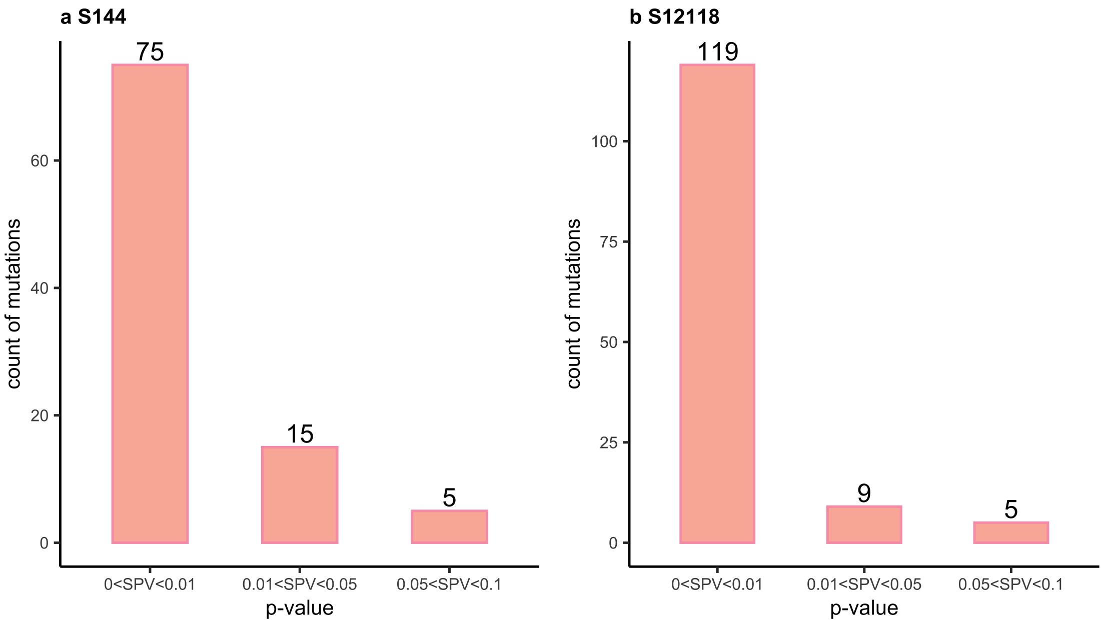

Fig S4: **Somatic variant p-values for mutations that were shared between WES and scRNA-seq data.** Somatic p-values (SPV) are grouped into three intervals: $[0, 0.01], [0.01, 0.05], [0.05, 0.1]$ for (**a**) subject S144 and (**b**) subject S12118.

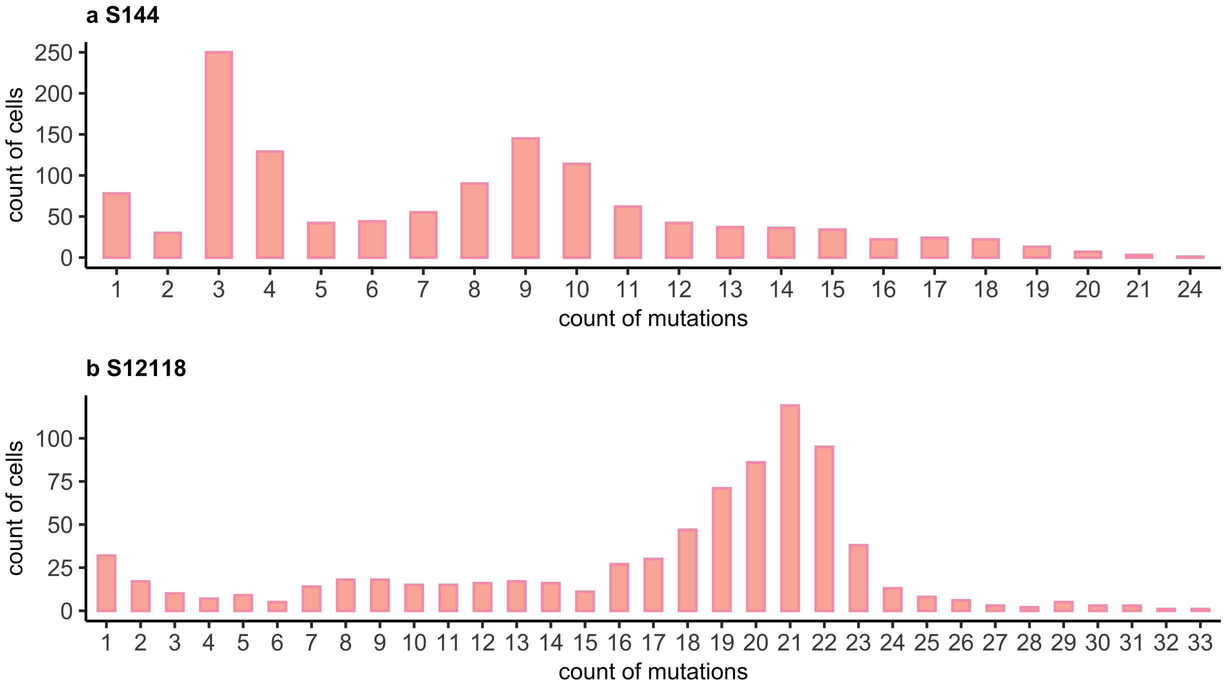

Fig S5: **Numbers of cells (y-axis) with specific mutation counts (x-axis)** for the mutations that were detected both in WES and scRNA-seq data for (**a**) subject S144 and (**b**) subject S12118.

a) S144

	C1	C2	C3	C4
C1	**0**	75	69	53
C2	78	**7**	39	39
C3	68	37	**7**	27
C4	57	40	24	**8**

b) S12118

	C1	C2	C3	C4
C1	**0**	89	112	94
C2	113	**40**	19	47
C3	100	49	40	**22**
C4	114	43	**10**	46

Fig S6: **Distances between the genotypes** corrected by CACTUS (x-axis) to the genotypes corrected by cardelino (y-axis), for subject S144 (**a**) and for subject S12118 (**b**). Both methods correct the genotypes of the clones provided as input. It may happen, that they infer similar genotypes by correcting initial genotypes of different input clones. This is the case for subject S12118, where genotype for input clone C3 corrected by CACTUS is the most similar to the genotype for input clone C4 corrected by cardelino, while genotype for clone C4 corrected by CACTUS is the most similar to the genotype for the input clone C3 corrected by cardelino. For a comparison of cell-to-clone and cluster-to-clone attachments by the two methods, we relabeled the clones so that the most similar genotypes between the two methods obtain the same labels (see the main text).
